# Evaluation of bactericidal effects of ultraviolet light C irradiation on cariogenic bacteria: An in vitro study

**DOI:** 10.1186/s12903-021-01767-x

**Published:** 2021-08-18

**Authors:** Moayyad Alharbi, Fahad Bakitian, Ali Alenezi

**Affiliations:** 1 Department of Preventive Dentistry, Ministry of Health, Tabuk, Saudi Arabia; 2 Department of Conservative Dentistry, College of Dentistry, Umm Al-Qura University, Makkah, Saudi Arabia; 3 Department of Prosthodontics, College of Dentistry, Qassim University, Buraydah, Saudi Arabia

**Keywords:** Ultraviolet light C, Dental caries, *Streptococcus mutans*, Bactericidal effect

## Abstract

**Backgrounds:**

Ultraviolet light C (UVL-C) irradiation has demonstrated an antimicrobial action against various pathogens. This study aimed to evaluate the bactericidal effect of UVL-C irradiation against cariogenic oral bacteria (*Streptococcus mutans*) in single layers and colonies grown on solid surfaces.

**Methods:**

Two different experiments were performed. In the first experiment, a single layer of *Streptococcus mutans* bacteria on agar plates was exposed to UVL-C irradiation at energies from 0 to 21 mWs/cm^2^. The second experiment was conducted to inhibit viability of bacterial colonies on solid surfaces. The samples were derived from saliva from a patient where bacteria were grown on plastic strips and then exposed to UVL-C. The highest energy was 1050 mWs/cm^2^.

**Results:**

Exposure to 21 mWs/cm^2^ was bactericidal in single layers of *Streptococcus mutans*. The result for bacterial colonies on solid surfaces indicated only a bacteriostatic effect, even at energies of 1050 mWs/cm^2^.

**Conclusions:**

Ultraviolet light C exhibits bactericidal effects on single layers of *Streptococcus mutans* but has a limited effect on bacterial colonies in a biofilm. It is a matter of debate whether these in vitro results would have the same effect in clinical setting.

## Background

Dental caries is the most common non-communicable oral disease with the highest prevalence globally [1]. (WHO) Global Oral Health revealed that 60–90% of school children and nearly 100% of the adult population have tooth cavities [2]. Dental caries is a multifactorial disease leading to localized destruction of tooth hard tissues [3]. This localized tooth destruction is caused by organic acids produced from cariogenic bacteria in the dental biofilm through metabolizing the fermentable carbohydrates [4]. When the dental biofilm is changed by acidity and nutrients of the oral environment, dental caries is developed [5]. The dental biofilm comprises complex microbial species embedded as colonies in the organic matrix [6, 7]. *Streptococcus mutans* (*S. mutans*) exist among the aggregated microbes in the dental biofilm, and it is considered the main pathogenic bacteria that cause dental caries [5].

For many decades, researchers have tried to find various methods to prevent dental caries. Using antibacterial agents, such as sodium fluoride, kanamycin, vancomycin, and chlorhexidine, has been proven to be excellent preventive methods because of the ability to diminish cariogenic bacteria [8–10]. However, the challenge of using these agents is the ability to reduce the number of cariogenic bacteria in the dental biofilm without affecting the ecological balance of oral flora [11]. Thus, the shifting toward new technologies for preventing dental caries is very prominent. Previous studies have evaluated the effectiveness of using ultraviolet light (UVL) irradiation on reducing the cariogenicity of some bacteria that cause dental caries [12–14]. This photodynamic technology in preventing dental caries was used first by Downes and Blunt in 1877 [15]. In dentistry, UVL irradiation has not been thoroughly investigated, and only a few experiments have been conducted. The first investigation of UVL irradiation effects on the dental plaque was in 1979 by Orstavik and Ruangsri [12]. In that study, small pieces of bovine enamel were mounted on the patient's mandibular molar by the orthodontic appliance to growth human oral bacteria. The specimens were taken out and exposed to UVL irradiation. The result was a reduction in plaque compared to the control group. In another study, absorption of UVL irradiation by a bacterial suspension was shown to prevent the effect on single layers of bacteria on agar [13, 16]. The UVL irradiation was used to kill various oral bacteria such as *Fusobacterium nucleatum*, *Lactobacillus brevis*, *Porphyromonas gingivalis*, *Enterococcus faecalis*, and *Streptococcus sanguinis* [13]. Relatively low energy levels of UVL irradiation were effectively used on single layers.

The UVL irradiation has three prime wavelengths: UVL-A (315–400 nm), UVL-B (280–315 nm), and UVL-C (100 to 280 nm). The specific type of UVL that affects microorganisms is UVL-C. The mechanism of action of UVL-C is to inactivate DNA replication by absorption of UVL, causing a photochemical reaction in the DNA chain that inhibits DNA replication [17]. Humans are not typically exposed to UVL-C because the ozone layer absorbs all UVL-C rays [18]. The mercury vapour lamp can generate this type of irradiation. Since UVL-C irradiation inhibits the growth of microorganisms, it might be interesting to investigate its effect in a biofilm model of cariogenic bacteria. It is of interest to evaluate the effect of UVL-C irradiation on both single layers of bacteria and bacterial colonies in a biofilm model. Previous studies have investigated the effect of UVL on single layers of bacteria and bacteria biofilms, and the main findings were that UVL has more impact on single layers of bacteria compared to bacteria in biofilms [12, 19]. However, no specific experiments have demonstrated how *S. mutans* cells resist UVL-C in a single layer or biofilm contexts. If the method is effective on bacterial colonies in a biofilm on a solid surface, it might indicate effectiveness in the oral cavity or even within an incipient caries lesion.

There is, however, only limited evidence on the effectiveness of UVL-C irradiation on cariogenic bacteria in a biofilm model. It would be useful to develop an uncomplicated method to evaluate the antibacterial effect of UVL-C irradiation on cariogenic bacteria in both single layers and colonies in a biofilm model. Therefore, the purpose of this in vitro study was to evaluate the bactericidal effect of UVL-C irradiation against cariogenic bacteria in single layers and colonies under the hypothesis that UVL-C irradiation can inhibit the growth of cariogenic bacteria.

## Methods

### Experiment 1: Single layer

**Fig. 1:**
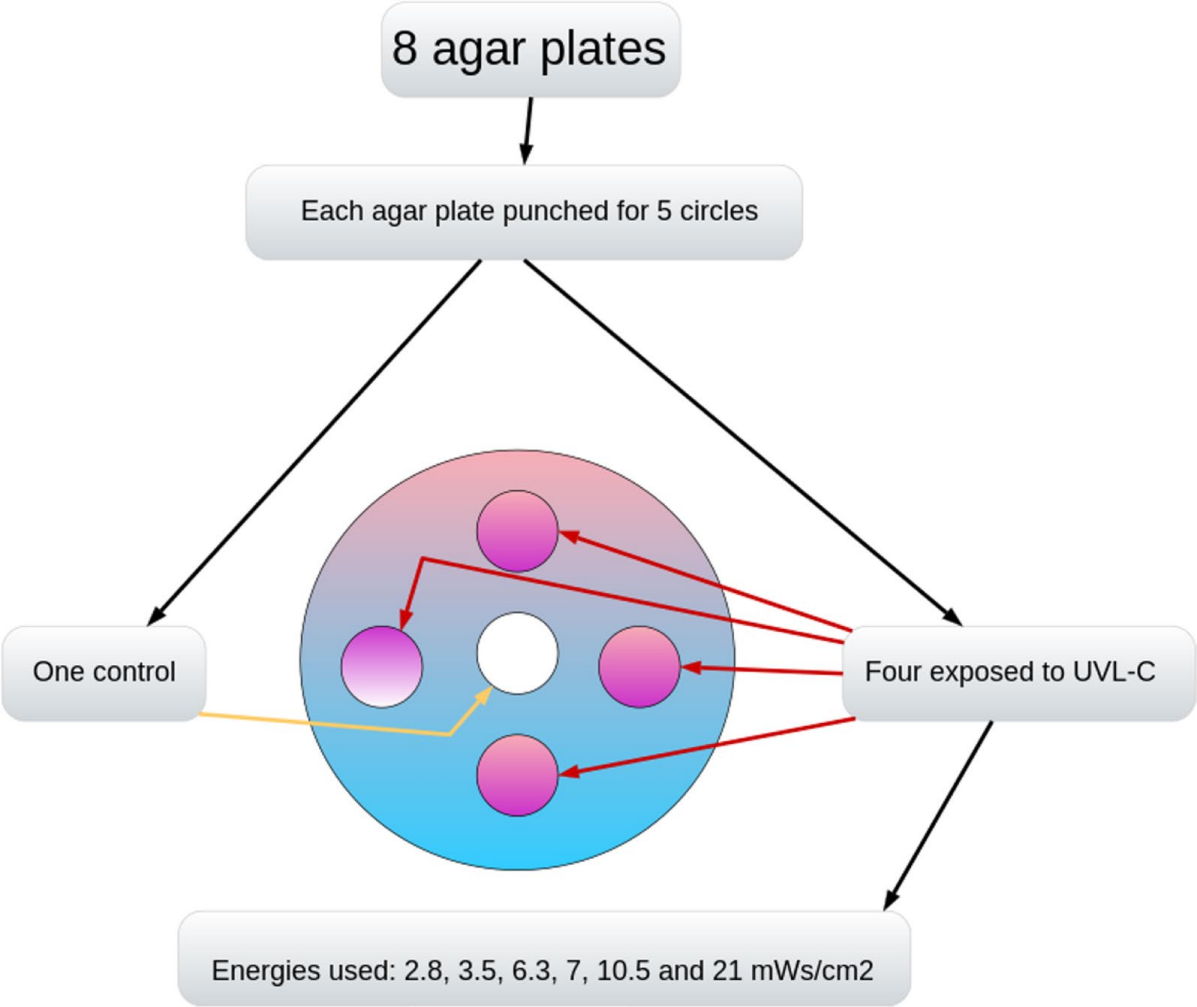
A model on agar plate demonstrates the exposed and unexposed area on single layer bacteria

*Streptococcus mutans* (NCTC 10,449), originally a clinical isolate, was inoculated into Todd Hewitt broth (TH) (Difco) and incubated for 24 h in the incubator at 37°. By serial dilution in TH, the bacteria were diluted to 10–4 and 10–8. 100 µl of bacterial dilutions were evenly distributed on MS-agar (Difco Mitis Salivarius Agar) plates using sterile glass beads. For each dilution, a total of eight agar plates were prepared by punching circles with a diameter of 1.5 cm in five sites. The total number of punched circles in each agar plate was five, one of which was the control and four of which were exposed to UVL-C (Fig. 1).

The bacteria within the punched circles were exposed to UVL-C using a UV lamp (Utbyteslysrör UVC 24 W Cyklon, China), placed in a black container with an aperture of 1.3 cm. The restriction of the light beam was controlled with black paper. The distance from the light aperture was obtained using black tubes of the same diameter, but different length thus giving defined effects on a defined area of bacteria. The tubes also restricted the light path to prevent radiation of the operator. After pilot experiments, the following set-up was used. Bacteria were exposed to the following energies: 2.8, 3.5, 6.3, 7, 10.5 and 21 mWs/cm^2^, by changing the exposure time.

The effect was measured at the agar surface by a photometer (ILT77CE Germicidal UV-C Light Meter International Light Technologies, USA), and the energies of UVL-C were adjusted by the time of exposure.

All agar plates were incubated for 48 h at 37° C in 5% CO2 in N2, the agar plates were scanned in a (Hewlett packard scanjet 4c/T), and the colonies were counted by a custom-made computer program. A total of 39 exposed areas (with four replications for each energy) was thus analyzed.

### Experiment 2: Colonies

Four saliva samples were taken from one subject that was a known carrier of *mutans streptococci* by the use of plastic strips (Dentocult® SM Strip Mutans Oral Care, Japan) incubated for 48 h at 37 °C. This method is based on using a selective culture broth (containing sucrose and bacitracin) and the adherence of *mutans streptococci*.

**Fig. 2:**
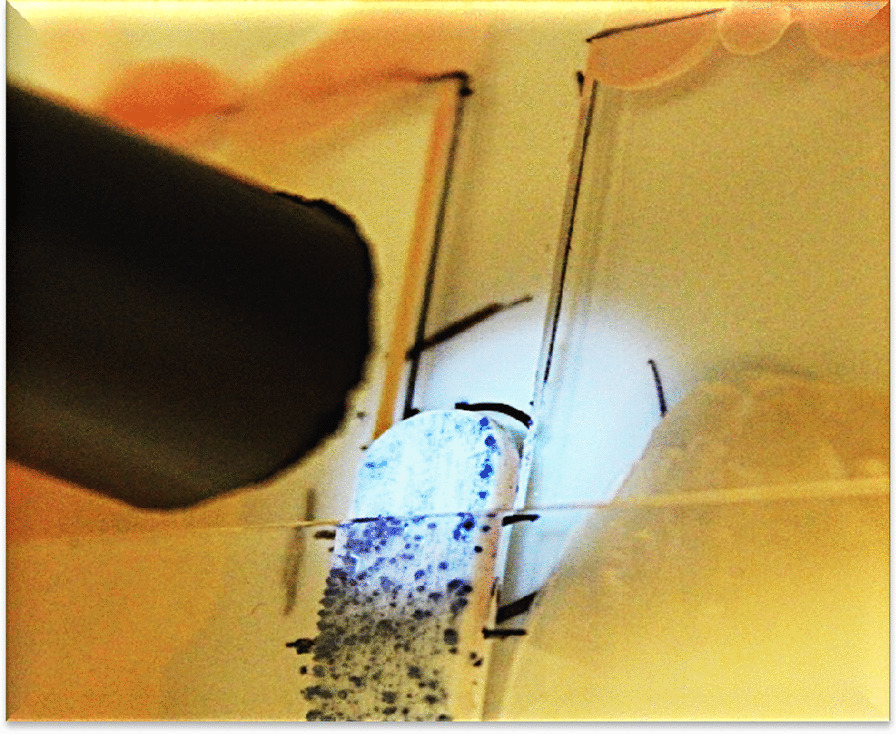
The colonies grew up onto the plastic strip. Half of the colonies were exposed to UVL-C and the other half were unexposed (covered with UV light protection)

The plastic strips were removed from the broth, and the excess broth was removed by touching a clean paper tissue. The strips were placed in a holder with the colony-covered side upwards. The holder's specifications were such that it allowed irradiation of half of the strip. Hence, half of the strip was exposed to UVL-C. The other half was covered with a UV-protective glass (O. Kindler GmbH, Germany) (Fig. 2). The distance from the light source was 2 cm, which was 3.5 mW/cm^2^. The bacteria were exposed to the following energies: 210, 420, 630, 1050 mWs/cm^2^.

Bacterial colonies from the exposed part were removed by a sharp, sterilized spatula with a safe margin not to include unexposed colonies. It was then transferred to a test tube containing fluid media and sterile glass beads. The colonies were dispersed using a Vortex mixer for 2 min, then serially diluted in TH to 10–3 and 10–4. 100 µl of bacterial dilutions were evenly distributed on MS-agar plates using sterile glass beads incubated for 48 h at 37° C in 5% CO_2_ in N_2_. The colonies were counted under a microscope at 2.5 magnification. A total of 5 plates was thus analyzed, examining all the energy levels. The output from the UV lamp was measured using the photometer. Measurements were performed at distances of 5.5 and 11.0 cm from the aperture.

## Results

### Inhibition of single layer bacterial growth on agar plates

**Fig. 3:**
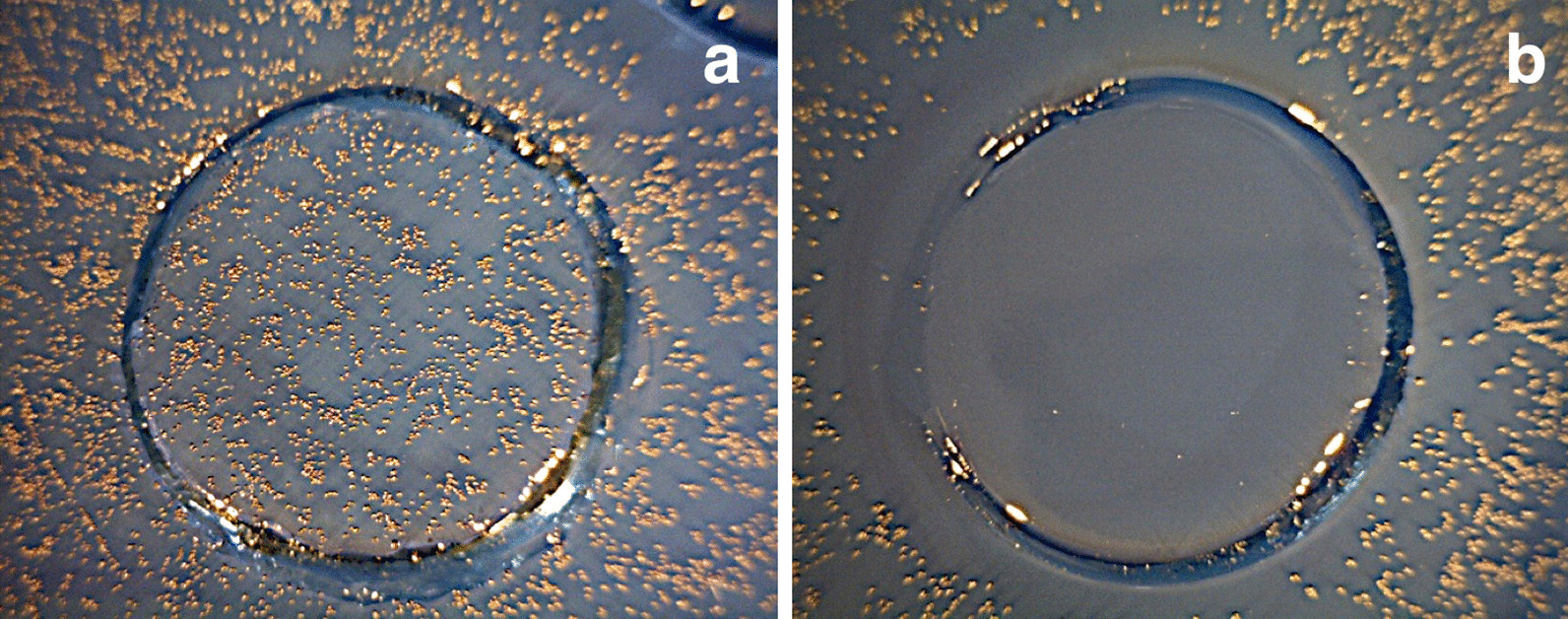
Control (**a**) and exposed (**b**) samples on single layer bacteria

**Fig. 4:**
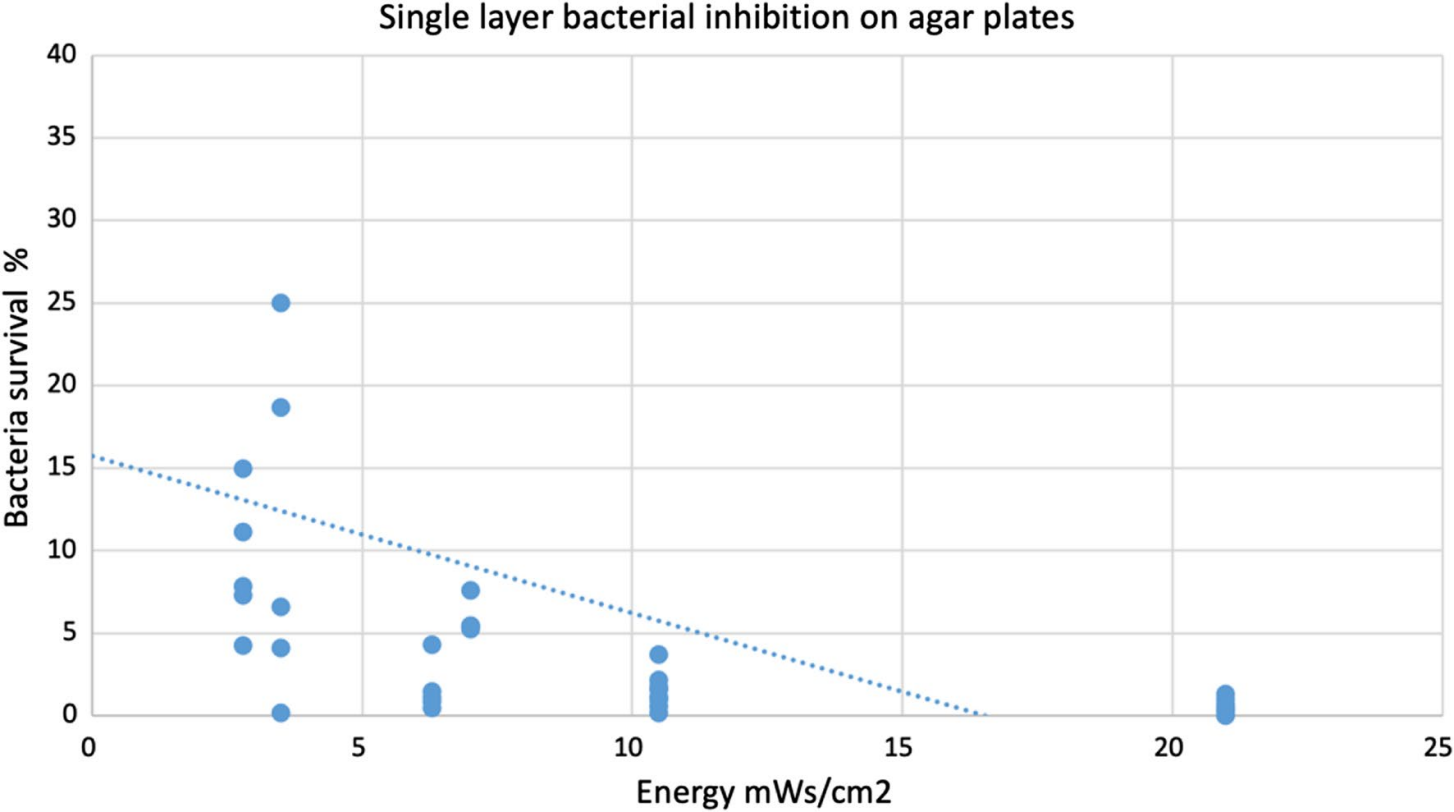
Survival rate of *S. mutans* strain 10,449 exposed to different energies of UVL-C. The blue dots demonstrate the percentage survival of bacteria on the exposed site relative to control. At 21 mWs/cm^2^, the bacterial survival was completely eliminated

Bacterial growth in single layers on agar plates was related to exposure to UVL-C (Fig. 3). Direct exposure of the *S. mutans* to UVL-C eliminated all the bacteria, even some bacteria outside the border determined in the experiment. The energy needed to kill all the bacteria was 21 mWs/cm^2^; more than 95% of the bacteria were killed at 11 mWs/cm^2^ (Fig. 4). Besides, the survival rate of the bacteria increased gradually as the energy decreased, from 7 mWs/cm^2^ until it became 100% survival at 0 mWs/cm^2^.

### Inhibition of bacterial colony growth on solid surfaces

**Fig. 5:**
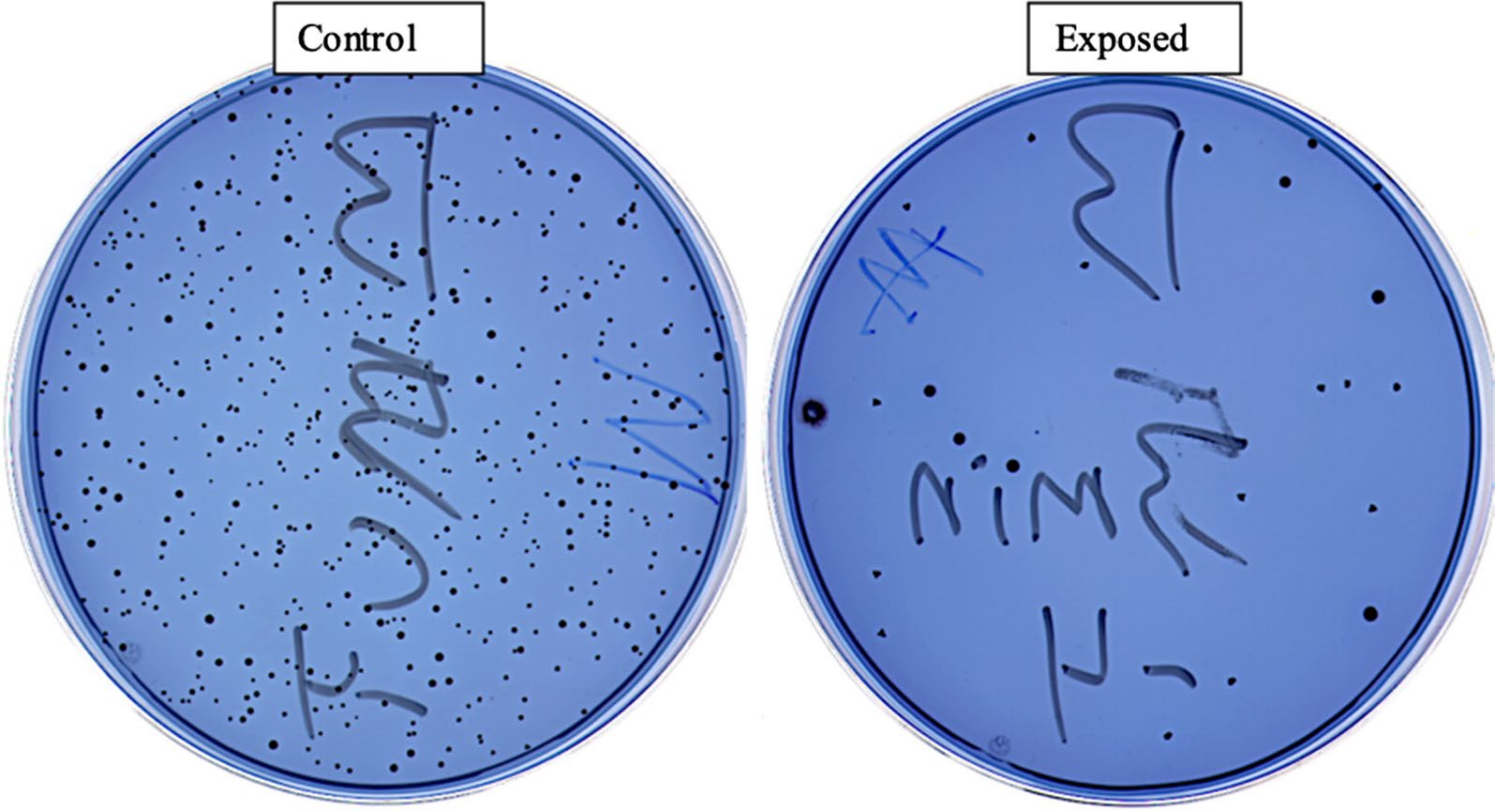
Growth after exposure of colonies. The effect of UVL-C on exposed colonies compared to unexposed colonies. The exposed colonies were significantly affected by UVL-C

**Fig. 6:**
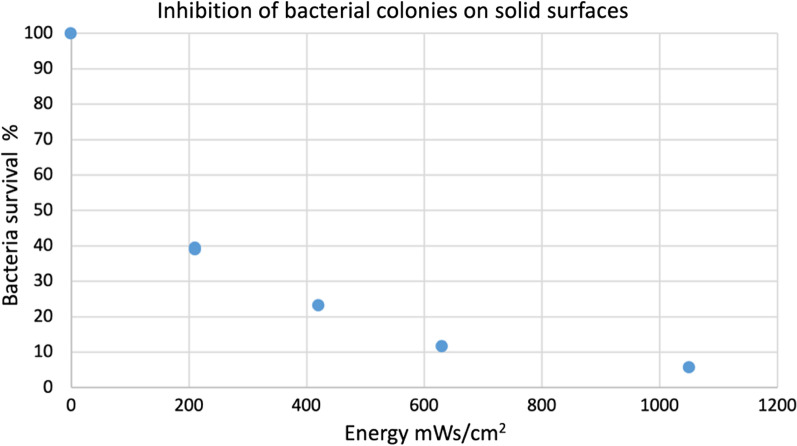
The relationship between surviving bacteria and energy levels. Between 300 and 1000 mWs/cm^2^, the survival rate was between 10 and 30%. There was bacteriostasis at the highest energy (1050 mWs/cm^2^), and the survival was around 6%

Direct exposure of the *S. mutans* colonies to UVL-C eliminated most of the viability of the bacteria compared to a control. The energy of 1050 mWs/cm^2^ was bacteriostatic (Figs. 5 and 6).

## Discussion

In this laboratory study, the aim was to evaluate the antibacterial effect of UVL-C on one group of cariogenic bacteria, *mutans streptococci*, using two different in vitro experiments to find a new method to control the viability of these organisms in the oral cavity.

UV light, in general, is harmful to human cells. The effects of UVL-A and UVL-B irradiations are well known, and many studies show their side effects on humans [20]. The biological effect is apparent, especially on the eyes and skin [20, 21]. The effect of UVL irradiation depends on the duration of exposure, and it can be classified into acute and chronic effects [22–24]. Some in vitro studies showed that exposure to UVL-B irradiation can cause damage to human corneal epithelial cells [25]. Furthermore, exposure to UVL-A and UVL-B were claimed to be responsible for the development of skin cancers in animals and in immunosuppression in humans [20]. Basal cell carcinoma and malignant melanoma are common forms of these cancers [26]. Meanwhile, UVL-C is believed to have a negligible adverse effects human health [20].

The main findings of this in vitro study were that single layers of *mutans streptococci* were easily killed at low energies of UVL-C irradiation, but in colonies, *mutans streptococci* were less susceptible to UVL-C. However, a bacteriostatic effect could be seen for bacterial colonies. The results were in line with other published results for other bacteria using other models comparing biofilm and single layer bacteria [13, 19, 27, 28]. However, this is the first demonstration of the effect on *mutans streptococci* using an uncomplicated model for biofilm effects using the plastic strip (Dentocult® SM Strip Mutans Oral Care, Japan), a method designed initially for chair-side measurement of *mutans streptococci*.

### Inhibition of single layer bacteria on agar plates

The UVL-C was able to kill the bacteria on the agar plates. A total bactericidal effect against this laboratory strain of *S. mutans* was achieved in the single layer bacteria assay. The effect of radiation was noticeable even using a small amount of energy. The highest energy used was 21 mWs/cm^2^; at this point, no bacteria tolerated the radiation. The energies between 3 and 11 mWs/cm^2^ showed a survival rate of less than 30% at 3 mWs/cm^2^. This implies that UVL-C can kill single layer bacteria and that the energy required for bactericide is low. In a single layer of bacteria, there is no energy absorption before the UVL-C reaches the bacteria; however, in a biofilm with multi-layers of bacteria and bacterial products such as polysaccharides energy absorption occurs. The variation in survival rates at low energies in the current study may be due to small variations in exposure time and that the bacterial cells were in various stages of growth. Future studies are needed to replicate this study's findings.

The results from the present study on single layers of *mutans streptococci* were comparable to the findings reported in other studies where the antibacterial effect and varying intensities of UVL-C on *non-mutans streptococci* species have been proven [13]. Moreover, the findings of this study were similar to those found by previous study on exposure to UVL-C of single layers of *Fusobacterium nucleatum*, *Lactobacillus brevis*, *Porphyromonas gingivalis*, *Enterococcus faecalis* and *S. sanguinis *[13]. In that study, UVL-C eliminated 99.9% of the bacteria at energies of 6–7 mWs/cm^2^ 20 [13]. Interestingly, the energy needed to kill the bacteria differed between species [29]. For example, *S. sanguinis* was eliminated at 2 mWs/cm^2^, while 7 mWs/cm^2^ was needed to kill Lactobacillus brevis. Notably, the energies required to kill the bacteria in that study are in the same range of energies as those required for killing *S. mutans* in the present study. Furthermore, the present study was carried out on vegetative cells, which could be expected to be more sensitive than spores due to the interferences of UVL-C on DNA replication [17]. A further contribution to the literature is that the current study's method was uncomplicated, with the main components being a UVL-C lamp, *S. mutans*, agar, TH, and black tubes of varying lengths. The results indicate that the method is useful and easy to use for measuring effects of UVL-C on *mutans streptococci* and that UVL-C is useful as a bactericide of *mutans streptococci* at specific intensities.

### Inhibition of bacterial colonies on solid surfaces

The second experiment aimed to investigate UVL-C exposure on *mutans streptococci* growth in colonies that mimic oral plaque. Colonies were expected to require higher energy to reduce bacterial viability compared to a single layer of bacteria. As expected, at energies of 200, 400, and 500 mWs/cm^2^, the survival of exposed bacteria was only partially reduced. Between 300 and 1000 mWs/cm^2^, the survival rate decreased from 30 to 10%. Besides, at 1050 mWs/cm^2^, which was the highest energy, survival was around 6%.

The results of this study should be considered in light of its limitations. It is possible that the surviving bacteria can be explained by the fact that the UVL-C could not penetrate all the layers of *mutans streptococci* due to the colony thickness. Future studies should examine this variable more closely. It is also known that the bacteria in biofilms are more resistant to antimicrobials [30]. This is likely due to the complex community of the biofilm and the production of an exopolysaccharide matrix [31]. The antimicrobial agents have limited effects due to a barrier they develop that limits their diffusion into a biofilm. This barrier prevents antimicrobials from penetrating one cell after another [30]. Biofilms are useful in this area of study because they mimic the complex environment of the oral cavity [32]. To be applied to the dentistry, studies that show bactericidal effects on single layer bacteria should be replicated using biofilm. The implications of which were demonstrated by the current study and a previous study of the antibacterial effects of chlorhexidine on biofilm, which was resistant, while single-layer bacteria was easy to kill [33]. The results of this study are similar to those found in previous studies [12, 13]. Metzger et al. showed that a bacterial suspension could prevent the effect of UVL-C on single layers of bacteria on agar [13]. Meanwhile, Orstavik and Ruangsri showed that although UVL-C significantly reduced the number of bacteria not all bacteria in the biofilm could be killed, and UVL-C could not penetrate the last layer of plaque [12]. The collective results suggest limitations of using UVL-C for a bactericide on multiple and complex layers of bacteria that mimic those in the oral cavity. In this experiment, it was important to control the size of the colonies, removal of equal amounts of bacteria from the plastic strips, and contamination of samples. The results indicate that those factors were under control, due to the relatively low variation of survival at each energy, except the very low energies. The main limitation of this study was that the UVL-C lamp produced energy limited to 24 W. Future studies should examine the effects of higher intensities on bacteria in the biofilm. In terms of generalizing the results, it should consider that this is an in vitro study, and results might not represent the clinical situation, where the oral biofilm is far more complex and influenced by various factors. Also, considering side effects, further investigation is essential for determining its use in humans.

The idea behind investigating UVL-C on a single layer of *S. mutans* was primarily to establish whether the microorganism was sensitive to UVL-C and at what energy levels. This study also provides data for comparison to other types of oral bacteria used in previous studies. The results from the present study indicate that UVL-C has an antibacterial effect on a single layer of *S. mutans* and a bacteriostatic effect on plaque.

Dental caries is a bacterial disease that theoretically can be eliminated by an antibacterial agent. According to the results of this study, the clinical indication of using UVL-C irradiation on caries lesion might be a promising method. For example, UVL-C acting on a superficial layer of soft caries in buccal and labial surfaces may reduce their bacterial load. However, this method cannot be applied alone and must be used as complementary to other treatments that help prevent the disease's progression. The dental application of UVL-C is a promising way that could help to reduce the number of bacteria in plaque on the teeth or during orthodontic treatment.

## Conclusion

Ultraviolet light-C can exhibit a bactericidal effect to single layer *Streptococcus mutans* but has a limited effect on bacterial colonies in a biofilm model. It is a matter of debate whether these in vitro results would have the same effect in the clinical setting. Thus, further studies in vitro and in vivo are needed to evaluate the effect of Ultraviolet light-C in reducing the number of bacteria on teeth surface.

## Data Availability

The datasets used and/or analyzed during the current study are available from the corresponding author on reasonable request.

## Abbreviations

UVL: Ultraviolet light
*S. mutans*: *Streptococcus mutans*

## References

[1] World Health Organization. Sugars and dental caries. World Health Organization; 2017.

[2] Petersen PE, Bourgeois D, Bratthall D, Ogawa H (2005). Oral health information systems-towards measuring progress in oral health promotion and disease prevention. Bull World Health Organ.

[3] West NX, Joiner A (2014). Enamel mineral loss. J Dent.

[4] Paes Leme AF, Koo H, Bellato CM, Bedi G, Cury JA (2006). The role of sucrose in cariogenic dental biofilm formation–new insight. J Dent Res.

[5] Gross EL, Beall CJ, Kutsch SR, Firestone ND, Leys EJ, Griffen AL (2012). Beyond Streptococcus mutans: dental caries onset linked to multiple species by 16S rRNA community analysis. PLoS ONE.

[6] Zijnge V, van Leeuwen MBM, Degener JE, Abbas F, Thurnheer T, Gmür R (2010). Oral biofilm architecture on natural teeth. PLoS ONE.

[7] Fiorillo L, Cervino G, Laino L, D'Amico C, Mauceri R, Tozum TF, et al. *Porphyromonas gingivalis*, periodontal and systemic implications: a systematic review. Dent J (Basel). 2019;7(4). 10.3390/dj7040114 PMC6960968 31835888

[8] Chen L, Suh BI, Yang J. Antibacterial dental restorative materials: A review. Am J Dent. 2018;31(Sp Is B):6b-12b. 31099206

[9] Autio-Gold J (2008). The role of chlorhexidine in caries prevention. Oper Dent.

[10] Zhang Q, Van Palenstein Helderman WH, Van't Hof MA, Truin G-J (2006). Chlorhexidine varnish for preventing dental caries in children, adolescents and young adults: a systematic review. Eur J Oral Sci.

[11] García-Godoy F, Hicks MJ (2008). Maintaining the integrity of the enamel surface: The role of dental biofilm, saliva and preventive agents in enamel demineralization and remineralization. J Am Dent Assoc.

[12] Orstavik D, Ruangsri P (1979). Effects of bactericidal treatments on bacterial adherence and dental plaque formation. Scand J Dent Res.

[13] Metzger Z, Dotan M, Better H, Abramovitz I (2007). Sensitivity of oral bacteria to 254 nm ultraviolet light. Int Endod J.

[14] Uchinuma S, Shimada Y, Matin K, Hosaka K, Yoshiyama M, Sumi Y (2019). Effects of UVB and UVC irradiation on cariogenic bacteria in vitro. Lasers Med Sci.

[15] Downes A, Blunt TP (1877). The Influence of Light upon the Development of Bacteria. Nature.

[16] Pattison DI, Davies MJ (2006). Actions of ultraviolet light on cellular structures. EXS.

[17] Setlow P (2001). Resistance of spores of *Bacillus* species to ultraviolet light. Environ Mol Mutagen.

[18] Diffey BL (2002). Sources and measurement of ultraviolet radiation. Methods.

[19] Thai TP, Keast DH, Campbell KE, Woodbury MG, Houghton PE (2005). Effect of ultraviolet light C on bacterial colonization in chronic wounds. Ostomy Wound Manage.

[20] Gallagher RP, Lee TK (2006). Adverse effects of ultraviolet radiation: a brief review. Prog Biophys Mol Biol.

[21] Yam JC, Kwok AK (2014). Ultraviolet light and ocular diseases. Int Ophthalmol.

[22] Young AR (2006). Acute effects of UVR on human eyes and skin. Prog Biophys Mol Biol.

[23] De Fabo EC (2005). Arctic stratospheric ozone depletion and increased UVB radiation: potential impacts to human health. Int J Circumpolar Health.

[24] Milon A, Sottas P-E, Bulliard J-L, Vernez D (2007). Effective exposure to solar UV in building workers: influence of local and individual factors. J Eposure Sci Environ Epidemiol.

[25] Youn HY, McCanna DJ, Sivak JG, Jones LW (2011). In vitro ultraviolet-induced damage in human corneal, lens, and retinal pigment epithelial cells. Mol Vis.

[26] Armstrong BK, Kricker A (1993). How much melanoma is caused by sun exposure?. Melanoma Res.

[27] Nicholson WL, Schuerger AC, Setlow P (2005). The solar UV environment and bacterial spore UV resistance: considerations for Earth-to-Mars transport by natural processes and human spaceflight. Mutat Res.

[28] Dai T, Vrahas MS, Murray CK, Hamblin MR (2012). Ultraviolet C irradiation: an alternative antimicrobial approach to localized infections?. Expert Rev Anti Infect Ther.

[29] D'Amico C, Fiorillo L, Surace G, Cervino G, Cicciù M (2021). In-vitro study on the effectiveness of microwave sterilization in odontostomatology. Minerva Dent Oral Sci.

[30] Mah TF, O'Toole GA (2001). Mechanisms of biofilm resistance to antimicrobial agents. Trends Microbiol.

[31] Fiorillo L (2020). We do not eat alone: formation and maturation of the oral microbiota. Biology (Basel).

[32] Thomas JG, Nakaishi LA (2006). Managing the complexity of a dynamic biofilm. J Am Dent Assoc.

[33] Bonez PC, Dos Santos Alves CF, Dalmolin TV, Agertt VA, Mizdal CR, Flores Vda C (2013). Chlorhexidine activity against bacterial biofilms. Am J Infect Control.

